# Polyglycolic Acid-Reinforced Sutures for Alveolar-Bronchiole Level Fistula Closure During Acute Empyema Surgery

**DOI:** 10.1093/icvts/ivaf188

**Published:** 2025-08-11

**Authors:** Junichi Murakami, Toshiki Tanaka, Mototsugu Shimokawa, Sota Yoshimine, Naohiro Yamamoto, Hiroshi Kurazumi, Kimikazu Hamano

**Affiliations:** Division of Chest Surgery, Department of Surgery and Clinical Science, Yamaguchi University Graduate School of Medicine, Ube, Yamaguchi 755-8505, Japan; Division of Chest Surgery, Department of Surgery and Clinical Science, Yamaguchi University Graduate School of Medicine, Ube, Yamaguchi 755-8505, Japan; Department of Biostatistics, Yamaguchi University Graduate School of Medicine, Ube, Yamaguchi 755-8505, Japan; Division of Chest Surgery, Department of Surgery and Clinical Science, Yamaguchi University Graduate School of Medicine, Ube, Yamaguchi 755-8505, Japan; Division of Chest Surgery, Department of Surgery and Clinical Science, Yamaguchi University Graduate School of Medicine, Ube, Yamaguchi 755-8505, Japan; Division of Chest Surgery, Department of Surgery and Clinical Science, Yamaguchi University Graduate School of Medicine, Ube, Yamaguchi 755-8505, Japan; Division of Chest Surgery, Department of Surgery and Clinical Science, Yamaguchi University Graduate School of Medicine, Ube, Yamaguchi 755-8505, Japan

**Keywords:** acute empyema, alveolar fistula, video-assisted thoracoscopic surgery, polyglycolic acid, suture techniques

## Abstract

**Objectives:**

Closure of alveolar-bronchiole level fistulas encountered during surgery for acute empyema is challenging due to compromised lung tissue, increasing the risk of persistent air leaks and infection. The use of polyglycolic acid (PGA)-reinforced sutures offers potential, but their application in infected fields is debated. This study evaluates the safety and efficacy of this technique for managing such intraoperative fistulas during video-assisted thoracoscopic (VATS) decortication.

**Methods:**

This single-centre retrospective study between 2017 and 2023 compared outcomes in patients undergoing VATS decortication for acute empyema. Patients requiring intraoperative closure of alveolar-bronchiole fistulas using PGA-reinforced sutures (Suture group, *N* = 7) were compared to those without identified fistulas (Control group, *N* = 14). Patients with post-resection empyema were excluded. The primary end-point was postoperative complications.

**Results:**

The Suture group had longer surgical durations (mean 139.3 vs 103.5 min, *P* < 0.01) and greater intraoperative blood loss (mean 192.0 vs 58.1 g, *P* = 0.04) compared to Controls. All identified air leaks in the Suture group were successfully sealed intraoperatively, confirmed by water-seal test and intraoperative confirmation test. Crucially, the Suture group experienced significantly fewer postoperative complications (Clavien-Dindo ≥ Grade 2) (0/7 [0%] vs 8/14 [57.1%], *P* = 0.01). No re-exacerbations or re-operations occurred in the Suture group, compared to 4 each in controls. Postoperative inflammatory markers decreased similarly in both groups (*P* > 0.05).

**Conclusions:**

PGA-reinforced suturing for alveolar-bronchiole fistulas during acute empyema surgery appears feasible and safe. While fewer complications were noted in the Suture group, this small, non-randomized study precludes definitive conclusions on efficacy. Larger prospective studies are warranted.

## Graphical Abstract

**Figure:**
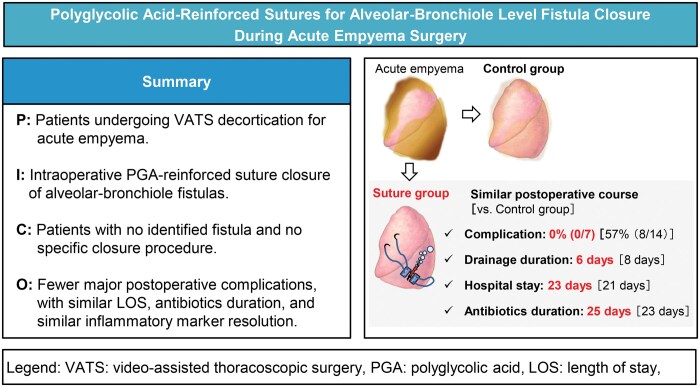

## INTRODUCTION

Acute intrathoracic infections, especially empyema, are serious conditions that require immediate and aggressive treatment.^1^ Surgical management, often considered the standard of care based on established guidelines and consensus,^2^^,^^3^ is frequently employed to address these infections. Thoracic surgeon occasionally encounters peripheral bronchopleural fistulas due to intraoperative manipulation and necrosis with infection. Closure of these fistulas is notoriously challenging because of the fragility of the parenchymal lung tissue. Inaction or failure to treat can lead to the persistence or recurrence of infection, potentially progressing to a chronic state.

Various techniques have been employed for fistula closure, including direct suturing, muscle flaps, and thoracoplasty, each with inherent limitations. Our group has previously developed and reported the efficacy of a method utilizing polyglycolic acid (PGA) pledgets and absorbable monofilament sutures for intraoperative air leak control after thoracoscopic pulmonary resection.^4^^,^^5^ Building upon our previous experience with the method, this study specifically focuses on their application for alveolar-bronchiole level fistula closure during surgery for acute empyema.

Despite their biocompatibility, using artificial medical materials such as PGA and absorbable monofilament sutures in infected surgical fields is still controversial.^6^^,^^7^ This highlights the need for rigorous evaluation. Therefore, the primary objective of this retrospective study is to describe our initial experience and to evaluate the feasibility and safety of PGA-reinforced sutures for alveolar-bronchiole level fistula closure during surgery for acute empyema. In this descriptive study, we report the outcomes of patients who underwent this procedure. The outcomes of a control group, who underwent the same primary surgery without identified fistulas, are presented as an institutional benchmark.

## PATIENTS AND METHODS

### Study design

This retrospective, single-centre, observational study was conducted at Yamaguchi University Hospital to evaluate the efficacy and safety of PGA-reinforced sutures for alveolar-bronchiole level fistula closure during surgery for acute empyema. The study protocol was approved by the Yamaguchi University Research Ethics Committee (IRB number: H2024-061; approval date: July 17, 2024), and the requirement for informed consent was waived due to the retrospective nature of the study.

### Study population

We retrospectively reviewed patients undergoing acute empyema surgery at Yamaguchi University Hospital (2017-2023). Inclusion required: (1) diagnosis of acute pleural infection based on ≥1 criterion (pleural fluid pH < 7.2; or glucose < 40 mg/dl [2.2 mmol/L] and lactate dehydrogenase (LDH) > 1000 IU/L if pH unavailable; or presence of pus/microorganisms; or observable pus in the pleural space^2^^,^^3^); (2) undergoing video-assisted thoracoscopic surgery (VATS) decortication; and (3) age ≥18 years. Exclusions were empyema after pulmonary resection, empyema originating from adjacent non-lung organ infection, or requirement for open window thoracotomy. Patients from this eligible cohort who did not have an intraoperatively identified fistula requiring closure constituted the control group.

### Data collection

Data were retrospectively collected from the medical records of eligible patients. Extracted data encompassed patient demographics, significant comorbidities, preoperative assessments (laboratory/microbiology results, RAPID score,^8^ Light classification,^9^ preoperative drainage, and timing to operation), operative details, postoperative course, and pertinent postoperative complications including recurrence, prolonged air leak, reoperation, and mortality.

### Surgical procedure and postoperative management

VATS decortication was performed under general anaesthesia and 1-lung ventilation via multiple ports (1-5 cm) without rib spreading, involving meticulous dissection of adhesions/septa, pus removal, and extensive saline irrigation. When the parietal pleura was markedly thickened and restrictive, a partial pleurectomy was also performed to ensure full lung re-expansion. After debridement, air leaks were identified using a water-seal test.^10^ Intraoperatively, ruptured lung abscesses were defined as air leaks accompanied by pus discharge from the lung parenchyma, whereas iatrogenic lung injuries were defined as air leaks without pus discharge. Identified alveolar-bronchiole fistulas were closed using PGA-reinforced 4-0 PDS II^®^ sutures (continuous overlock or horizontal mattress with 0.3 mm PGA pledgets), then further reinforced with 0.15 mm PGA felt and fibrin glue (**Video S1**). This technique was adapted from methods previously described for anatomical lung resection. No specific fistula closure was performed in the control group. Chest tubes were placed, and pneumostasis was confirmed intraoperatively using our established method.^10^

Postoperatively, chest tubes were connected to water-seal drainage with appropriate suction (typically −3 to −10 cmH_2_O based on surgeon preference and air leak presence). Initial broad-spectrum antibiotics were administered and adjusted based on culture results and clinical response, with de-escalation when appropriate. Chest tube removal followed standard criteria: minimal non-purulent drainage (<100 mL/day), absence of air leak, radiographic evidence of lung re-expansion, and overall infection control.

### Primary and secondary outcomes

The primary end-points of this study were short-term postoperative outcomes, specifically re-exacerbation, 90-day mortality, and the incidence of postoperative complications (grade 2 or higher on the Clavien-Dindo classification). Secondary end-points included surgical duration, intraoperative blood loss, duration of postoperative drainage, length of hospital stay, duration of postoperative antibiotic therapy, and the time course of laboratory data, including white blood cell (WBC) count, percentage of neutrophils in WBC, and C-reactive protein (CRP).

### Statistical analysis

Statistical analyses were performed using STATA 14 (Stata Corp., College Station, TX, USA). Continuous variables were expressed as medians [interquartile ranges (IQR)] and compared using the Mann-Whitney *U*-test. Categorical variables were presented as numbers and percentages and compared using Fisher’s exact test. Longitudinal changes for laboratory data (measured at preoperation, postoperative days 1, 3, and 7) were compared between groups using 2-way repeated-measures analysis of variance (ANOVA). A *P*-value < 0.05 was considered statistically significant.

## RESULTS

### Cohort description

**Figure 1.**
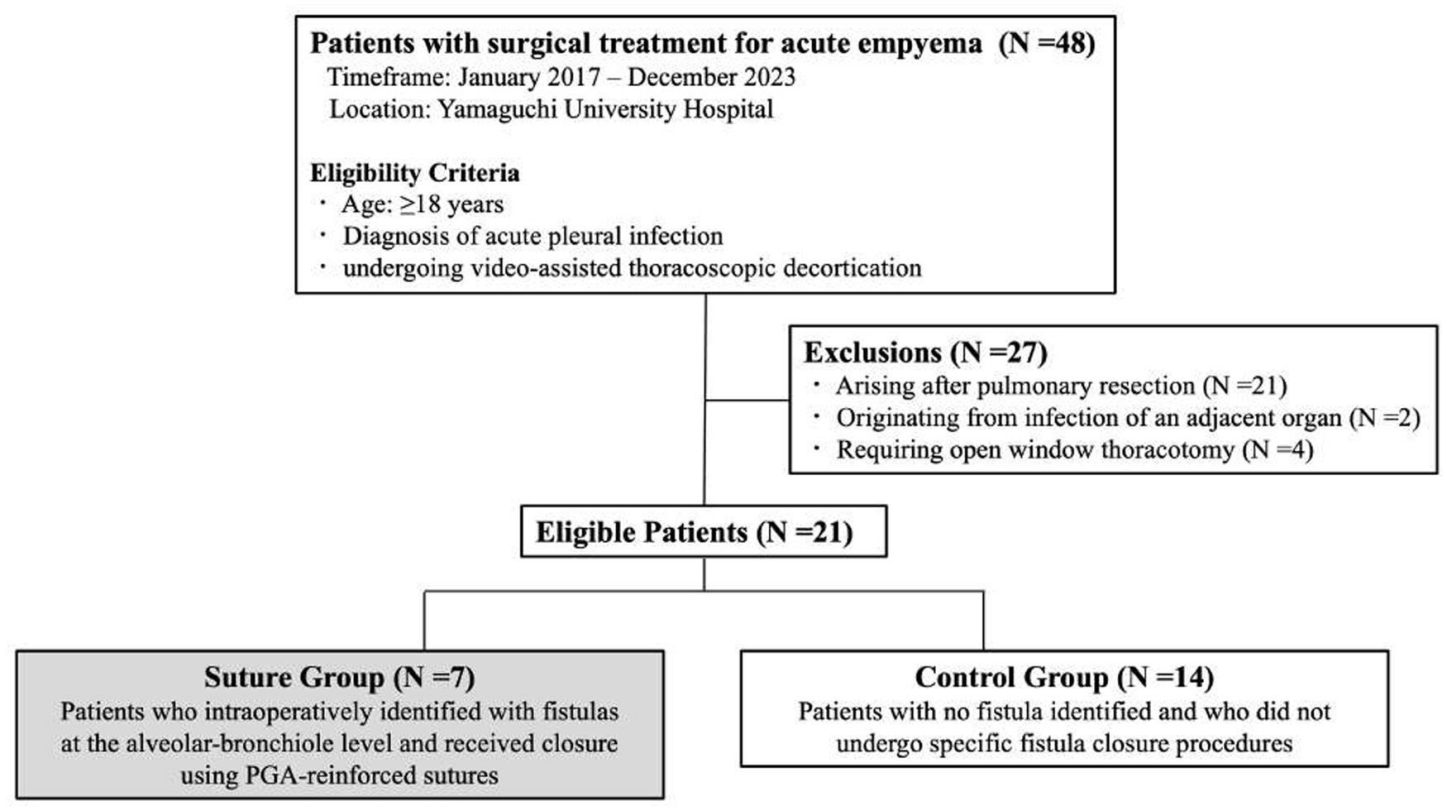
Flow Diagram of the Patient Selection Process. This figure illustrates the process of patient selection for the study. It shows the initial pool of patients undergoing acute empyema surgery, the application of exclusion criteria, and the final number of patients included in the suture and control groups

From January 2016 to December 2023, 48 patients underwent video-assisted thoracoscopic decortication for acute empyema at Yamaguchi University Hospital. Of these, 27 patients were excluded for the following reasons: empyema arising after pulmonary resection (*n* = 21), originating from an adjacent organ infection (*n* = 2), or requiring an open window thoracotomy (*n* = 4). Consequently, a total of 21 patients were identified for this study (**Figure 1**). Among these, 7 patients who underwent PGA-reinforced sutures for the closure of alveolar-bronchiole level fistulas comprised the suture group. In comparison, 14 patients with no fistula identified and did not receive specific fistula closure procedures made up the control group.

### Patients characteristics

As shown in **Table 1**, some imbalances in baseline characteristics were observed between the 2 groups; however, none of these differences were statistically significant.

**Table 1.** Baseline Characteristics of Patients in the Suture Group Compared to Those in the Control Group

		Overall	[IQR]	Suture group	[IQR]	Control group	[IQR]	*P*-value
		*N* = 21	(%)	*N* = 7	(%)	*N* = 14	(%)	
Age		72	[66-76]	72	[59-82]	72	[68-76]	0.76^a^
Gender	Male	16	(85.7)	6	(85.7)	10	(71.4)	0.62^b^
ECOG-PS	0/1/2/3	9/5/3/4		4/1/1/1		5/4/2/3		0.91^b^
Smoking history	Never/ever/current	7/12/2		2/4/1		5/8/1		1.00^b^
Anaemia	Hb <8.0	1	(4.8)	0	–	1	(7.1)	1.00^b^
Arrhythmia		3	(14.3)	1	(14.3)	2	(14.3)	1.00^b^
CKD	≥Grade 3b	4	(19.0)	1	(14.3)	3	(21.4)	1.00^b^
COPD		5	(23.8)	3	(42.9)	2	(14.3)	0.28^b^
CVD		2	(9.5)	1	(14.3)	1	(7.1)	1.00^b^
Dyslipidaemia		4	(19.0)	1	(14.3)	3	(21.4)	1.00^b^
Diabetes mellitus		5	(23.8)	2	(28.6)	3	(21.4)	1.00^b^
Haemodialysis		0	(0.0)	0	–	0	–	–
Hypertension		12	(57.1)	3	(42.9)	9	(64.3)	0.40^b^
IHD		1	(4.8)	0	–	1	(7.1)	1.00^b^
Immunosuppression agents		1	(4.8)	0	–	1	(7.1)	1.00^b^
Interstitial pneumonia		0	–	0	–	0	–	–
Liver dysfunction	≥Child B	2	(9.5)	0	–	2	(14.3)	0.53^b^
Malignancy treatment	≥5 years	1	(4.8)	0	–	1	(7.1)	1.00^b^
	<5 years	5	(23.8)	2	(28.6)	3	(21.4)	1.00^b^
Steroid medication		1	(4.8)	0	–	1	(7.1)	1.00^b^

Continuous variables were expressed as medians [interquartile ranges (IQRs)]. Categorical variables were presented as numbers and percentages.

a Compared by the Mann-Whitney *U*-test.

b Compared using Fisher’s exact test.

Abbreviations: CKD: chronic kidney disease; COPD: chronic obstructive pulmonary disease; CVD: cardio-vascular disease; ECOG-PS: European cancer organization group-performance status; IHD: ischaemic heart disease.

### Details on preoperative empyema

**Table 2** demonstrated details about the empyema before the operation in the suture group and the control group. The 2 groups were similar in many ways. Most patients in both groups had drainage before surgery. The table also shows the results of bacterial culture tests in each group. In the suture group, 2 types of bacteria were identified in 2 patients, while in the control group, 4 patients had 2 or more bacteria, including 3 in 2 patients. In the suture group, the most common bacteria was *Streptococcus intermedius* (found in 4 out of 7 patients). In the control group, the most common bacteria were *Streptococcus intermedius* (found in 3 out of 14 patients) and *Escherichia coli* (found in 3 out of 14 patients). Various other bacterial species were detected in the control group.

**Table 2.** Empyema Characteristics and Microbiological Findings in Suture Group Compared to Control Group

		Overall	[IQR]	Suture group	[IQR]	Control group	[IQR]	*P*-value
		*N* = 21	(%)	*N* = 7	(%)	*N* = 14	(%)	
Affected side	Right	11	52.4	2	28.6	9	64.3	0.183^a^
Diagnose to operation	Day	5	[3-10]	3	[1-11]	6	[3-10]	0.37^b^
Preoperative drainage	Yes	18	85.7	6	85.7	12	85.7	1.00^a^
Empyema stage	1/2/3	0/15/6		0/4/3		0/11/3		0.35^a^
RAPID score	1/2/3/4/5/6	1/3/3/3/8/3		0/0/2/2/3/0		1/3/1/1/5/3		0.28^a^
Light classification	3/4/5/6/7	1/3/9/6/2		0/0/3/2/2		1/3/6/4/0		0.23^a^
Bacterial culture test	Positive	18	(85.7)	5	(71.4)	13	(92.9)	0.25^a^
Number of identified bacterial species	0/1/2/3	3/13/2/3		2/4/1/0		1/9/2/2		0.66^a^
Identified bacterial species	*Enterococcus faecalis*	1	(4.8)	0	–	1	(7.1)	
	*Escherichia coli*	3	(14.3)	0	–	3	(21.4)	
	*Fusobacterium nucleatum*	1	(4.8)	0	–	1	(7.1)	
	*Fusobacterium varium*	1	(4.8)	1	(14.3)	0	–	
	*MRSA*	2	(9.5)	0	–	2	(14.3)	
	*MSSA*	1	(4.8)	1	(14.3)	0	–	
	*Parvimonas micra*	2	(9.5)	0	–	2	(14.3)	
	*Porphyromonas gingivalis*	1	(4.8)	0	–	1	(7.1)	
	*Pseudomonas aeruginosa*	2	(9.5)	0	–	2	(14.3)	
	*Streptococcus anginosus*	1	(4.8)	0	–	1	(7.1)	
	*Streptococcus constellatus*	2	(9.5)	0	–	2	(14.3)	
	*Streptococcus intermedius*	7	(33.3)	4	(57.1)	3	(21.4)	
	*Streptococcus lugdunensis*	1	(4.8)	0	–	1	(7.1)	

Continuous variables were expressed as medians [interquartile ranges (IQR)]. Categorical variables were presented as numbers and percentages.

a Compared using Fisher’s exact test.

b Compared by the Mann-Whitney *U*-test.

Abbreviations: *MRSA*: methicillin‐resistant *Staphylococcus aureus*; *MSSA*: methicillin‐susceptible *Staphylococcus aureus*.

### Surgical findings

**Table 3** provides detailed information about the surgical procedures performed on 7 patients with intraoperative air leaks caused by fistulas at the alveolar-bronchiole level in the suture group. The affected lobes included up to 2 lobes, and fistulas were identified in as many as 3 locations. The air leaks occurred for 2 primary reasons: lung injury during decortication in 5 patients and ruptured lung abscesses in 2 patients. These issues were addressed using either continuous overlock sutures, horizontal mattress sutures, or a combination of both, depending on the cleft’s shape and size, as determined by each surgeon. Notably, all patients experienced cessation of air leaks on the day of surgery. The surgical duration was longer for the suture group (131 [113-153] min) compared to the control group (96 [92-121] min, *P* = 0.014). The amount of blood loss during surgery tended to be greater in the suture group (130 [14-350] grams) compared to the control group (16 [5-80] grams, *P* = 0.073). No cases requiring intraoperative blood transfusion were observed in both groups.

**Table 3.** Individual Patient Data on Surgical Findings and Air Leak Closure in the Suture Group

Case	Age	Gender	PS	Affected lobe	Number of fistulas	Possible cause of air leaks	Method of fistula closure	Surgical duration (minute)	Intraoperative blood loss (gram)
1	72	male	1	LUL	1	lung injuries	1 COS	110	560
2	66	male	0	LUL	1	lung injuries	1 COS	119	14
3	82	male	3	RUL and RLL	3	lung injuries	2 COS and 1 HMS	142	130
4	58	male	0	LLL	2	lung injuries	3 HMS	113	350
5	83	male	2	LUL	1	lung injuries	1 HMS	131	220
6	59	female	0	LUL and LLL	2	rupture of lung abscesses	6 HMS	153	10
7	72	male	0	RUL	1	rupture of lung abscesses	2 HMS	207	60

Abbreviations: COS: continuous overlock suture; HMS: horizontal mattress suture; LLL: left lower lobe; LUL: left upper lobe; PS: performance status; RLL: right lower lobe; RUL: right upper lobe.

### Postoperative outcomes

Postoperative outcomes, detailed in **Table 4**, showed the Suture group experienced significantly fewer complications (Clavien-Dindo ≥ Grade 2) compared to the Control group. Notably, no re-exacerbations, re-operations, or drain reinsertions occurred in the Suture group, while these events were observed in several control patients. However, the duration of postoperative chest tube drainage, length of hospital stay, and duration of antibiotic treatment were comparable between the 2 groups. A 90-day mortality due to ARDS occurred in the Control group, with none in the Suture group.

**Table 4.** Postoperative Characteristics of Patients in the Suture Group Compared to Those in the Control Group

		Overall	[IQR]	Suture group	[IQR]	Control group	[IQR]	*P*-value
		*N* = 21	(%)	*N* = 7	(%)	*N* = 14	(%)	
Postoperative complication	CD ≥G2	8	(38.1)	0	–	8	(57.1)	0.019^a^
Re-exacerbation	Yes	4	(19.0)	0	–	4	(28.6)	0.26^a^
Re-operation	Yes	4	(19.0)	0	–	4	(28.6)	0.26^a^
Drain reinsertion	Yes	2	(9.5)	0	–	2	(14.3)	0.53^a^
Postoperative drainage	Day	5	[4-8]	5	[4-9]	5	[4-8]	0.73^b^
Postoperative hospitalization	Day	18	[14-28]	24	[14-28]	16	[8-28]	0.43^b^
Postoperative antibiotics administration	Day	20	[18-30]	25	[20-33]	19	[14-30]	0.28^b^
90-day mortality	Yes	1	(4.8)	0	–	1	(7.1)	1.00^a^
Discharged home	Yes	16	(76.2)	7	(100)	9^c^	(64.3)	0.123^a^

Continuous variables were expressed as medians [interquartile ranges (IQR)]. Categorical variables were presented as numbers and percentages.

a Compared using Fisher’s exact test.

b Compared by the Mann-Whitney *U*-test.

c Patients not discharged home (*n* = 5, all in the control group) were transferred to other hospitals for continued rehabilitation or convalescence.

Abbreviation: CD: Clavien-Dindo classification.

**Figure 2.**
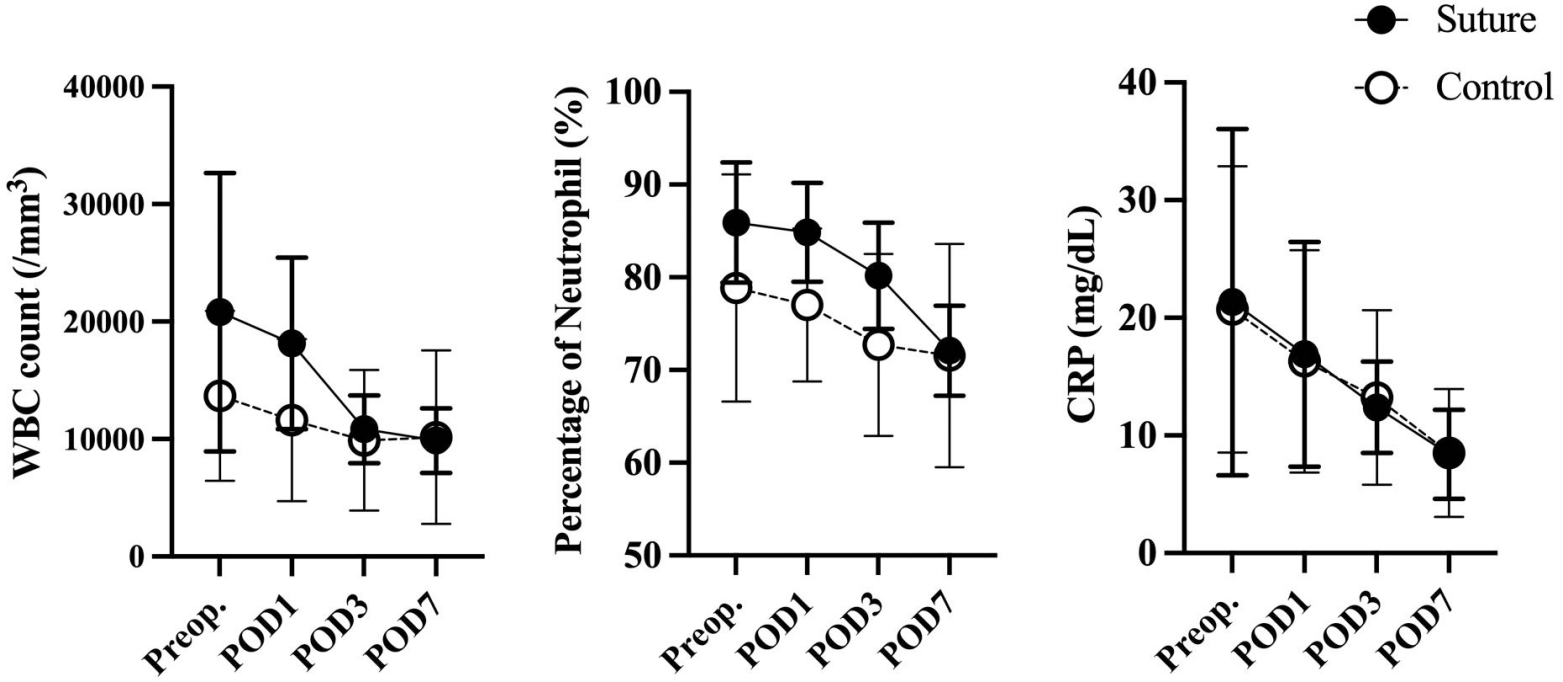
Longitudinal Changes for Laboratory Data Between Groups. This figure illustrates the changes in white blood cell (WBC) count, the percentage of neutrophils within the WBC count, and C-reactive protein (CRP) levels measured at preoperation, postoperative days 1, 3, and 7, over time for both the Stitch with PGA group and the Control group. The data are presented as the mean ± standard deviation. A 2-way repeated-measures analysis of variance (ANOVA) was used to conduct the statistical analysis

As shown in **Figure 2**, postoperative inflammatory markers (WBC count, neutrophil percentage, CRP levels) decreased over time following surgery in both groups. Importantly, 2-way repeated-measures ANOVA confirmed no statistically significant differences in the patterns of these inflammatory marker changes between the Suture and Control groups (WBC *P* = 0.21, Neutrophil% *P* = 0.124, CRP *P* = 0.98).

## DISCUSSION

This retrospective, single-centre study describes our initial experience with the use of PGA-reinforced sutures for alveolar-bronchiole level fistulas identified during VATS for acute empyema. This study was not designed to prove the superiority of the technique, but rather to report on its feasibility and safety in a challenging clinical setting. The outcomes of the control group are presented to provide a benchmark for the typical postoperative course at our institution for patients without this specific intraoperative complication. Intraoperatively, air leaks were sealed in all 7 patients of the Suture group. Although the Suture group had longer operations and greater blood loss, they experienced significantly fewer major postoperative complications, like re-exacerbation or reoperation, compared to controls. Importantly, systemic inflammation markers decreased similarly in both groups post-surgery, suggesting the suture material did not worsen infection.

Managing alveolar or bronchiole level fistulas during surgery for acute empyema presents a significant challenge. The lung tissue is often inflamed and fragile, making standard suturing techniques difficult and potentially causing further tissue damage.^11^^,^^12^ Persistent air leaks resulting from these fistulas are a well-recognized risk factor for prolonged hospital stays, persistent infection, and the need for further interventions.^13^^,^^14^ Various methods have been described for managing intraoperative air leaks or fistulas in non-infected surgical field, including simple sutures,^15^ application of surgical sealants,^16^^,^^17^ buttressing staple lines,^18^ or using tissue flaps for larger defects.^15^ However, the optimal direct approach specifically for small, peripheral fistulas encountered in the highly inflamed environment of acute empyema is less clear.

This study applies a technique our group previously developed for post-resection air leaks^4^^,^^5^ using thick PGA mesh pledgets with absorbable sutures to securely close fistulas where simple sutures might fail in fragile, compromised parenchyma. Its novelty lies in the specific application to alveolar-bronchiole fistulas during VATS for acute empyema, representing, to our knowledge, the first systematic evaluation in this challenging setting. This approach differs from managing large, chronic bronchopleural fistulas, often requiring complex procedures like muscle flaps or thoracoplasty.^15^^,^^19^^,^^20^ The current technique targets peripheral airway fistulas found unexpectedly during acute empyema surgery.

We chose to close all identified fistulas intraoperatively. While we acknowledge that minor leaks from decortication can resolve spontaneously, our policy in the infected setting of acute empyema is to be proactive. We believe any persistent air leak poses a substantial risk for re-exacerbation or chronic fistula. This rationale was applied to fistulas from both iatrogenic injuries (*n* = 5) and ruptured abscesses (*n* = 2) to minimize septic complications. Although limited resection is an important alternative for lung abscesses, it was deemed technically prohibitive in our cases. The severe inflammation made identifying clear margins for partial resection difficult, and dissection of the hilar structures for anatomical resection was considered hazardous. Therefore, our primary strategy was direct suture closure.

The most important finding is the apparent safety of using PGA-reinforced sutures. Achieving a durable seal to prevent a prolonged air leak, a major driver of morbidity, is the central technical goal of this approach. Despite the added complexity of fistula repair, the Suture group did not experience more complications than the institutional benchmark. While the observation of fewer complications is likely attributable to unanalysed confounders, this suggests the technique itself does not add significant morbidity. The comparable postoperative courses, including a prolonged hospitalization in both groups, likely reflect the time needed to resolve the underlying septic condition rather than the surgery itself. The PGA pledgets act as a buttress, distributing suture tension and preventing cut-through in compromised tissue, a common issue in empyema surgery.^21^ This concept is supported by canine experiments showing pledgets increase suture tensile strength.^22^ Such secure mechanical closure likely prevented persistent postoperative air leaks, known risks for infection persistence or recurrence,^23^^,^^24^ which aligns with our observation that all leaks in the Suture group resolved on the day of surgery.

A significant concern often raised is the use of synthetic, absorbable materials like PGA in an infected field, with some historical caution regarding potential nidus for infection or impaired healing.^6^^,^^7^ This study directly addressed this concern by evaluating outcomes in patients with active thoracic infection. The postoperative trends in blood inflammatory markers (WBC, neutrophils, CRP) were similar between the Suture and Control groups, suggesting that the PGA material did not provoke an excessive inflammatory response or worsen the underlying infection. PGA is known for its biocompatibility and predictable absorption profile,^25^ which might be advantageous over non-absorbable materials in an infected environment in term of biodegradability and resistance to pathogenic bacteria.^26^ The slightly longer operative times and increased blood loss in the Suture group are expected consequences of the additional time and manipulation required for fistula identification and repair. However, these did not translate into worse postoperative courses or the need for transfusions in this small cohort.

The relatively high complication rate (57.1%) in the Control group, despite not having an identified fistula needing suture, highlights the inherent risks associated with surgery for advanced acute empyema. The complication rate for acute empyema surgery is generally high, with variability depending on patient factors and comorbidities.^27^^,^^28^ Some patients in the Control group may have had severe parenchymal inflammation or remnant pyothorax cavity with septums, which could have contributed to complications such as re-exacerbation. This serves as a reminder of the general principle that achieving lung re-expansion and pleural space control by complete decortication is critical in all empyema patients.

The clinical implications of this study are that PGA-reinforced suturing represents a potentially valuable tool for surgeons managing challenging alveolar-bronchiole-level fistulas during acute empyema surgery. Effectively addressing these intraoperative leaks may reduce significant postoperative morbidity, such as empyema recurrence and the need for reintervention. Further research is needed to validate these initial findings. To accurately assess the effectiveness of this intervention, a future study should prospectively compare outcomes between patients with intraoperative leaks treated with PGA-reinforced sutures and a true control group of patients with similar leaks who receive no specific closure.

Several limitations warrant consideration when interpreting these results. The retrospective design carries inherent risks of bias, including selection bias and potential unmeasured confounders. As a single-centre university hospital study, generalizability may be limited due to differences in patient populations or surgical practices. This study’s primary limitations include its small sample size, which limits the ability to draw robust conclusions. Furthermore, the paradoxical finding of fewer complications in the Suture group lacks biological plausibility and is likely due to uncontrolled confounding factors, not evidence of the technique’s superiority. The comparison group, being non-randomized patients without identified fistulas, serves primarily as an institutional benchmark rather than a direct control for the intervention itself, allowing for potential confounding. Variability in antibiotic duration due to physician preference is another limitation. Additionally, variability among the multiple participating surgeons and the focus on short-term follow-up, which precludes assessment of long-term outcomes or potential late complications related to the PGA material, are further limitations. The predominance of upper lobe fistulas in the Suture group is likely a chance finding, due to the small cohort size, rather than the result of selection bias. The biologically implausible finding of fewer complications in the Suture group is not evidence of superiority, but likely reflects confounding factors such as the control group’s trends towards higher ECOG-PS scores and longer time-to-surgery.

## CONCLUSIONS

Based on our preliminary retrospective analysis, the use of PGA-reinforced sutures seems to be a feasible and potentially safe technique for managing alveolar-bronchiole level fistulas identified during VATS decortication for acute empyema. While the outcomes in this small cohort are encouraging, no definitive conclusions can be drawn from the current study regarding the efficacy or superiority of this method. Further research is essential to validate these initial observations.

## Supplementary Material

ivaf188_Supplementary_Data

## Data Availability

The datasets analysed in the current study are available from the corresponding author upon reasonable request.
